# Protective essential oil attenuates influenza virus infection: An in vitro study in MDCK cells

**DOI:** 10.1186/1472-6882-10-69

**Published:** 2010-11-15

**Authors:** Shuhua Wu, Krupa B Patel, Leland J Booth, Jordan P Metcalf, Hsueh-Kung Lin, Wenxin Wu

**Affiliations:** 1 Department of Respiratory Medicine, the First Affiliated Hospital of Soochow University, Suzhou, Jiangsu, 215006, PR China; 2 Department of Emergency and Critical Care, the Second Affiliated Hospital of Soochow University, Suzhou, Jiangsu, 215004, PR China; 3 Pulmonary and Critical Care Division, Department of Medicine, University of Oklahoma Health Sciences Center, Oklahoma City, OK 73104, USA; 4 Department of Microbiology and Immunology, University of Oklahoma Health Sciences Center, Oklahoma City, OK 73104, USA; 5 Department of Urology, University of Oklahoma Health Sciences Center, Oklahoma City, OK 73104, USA; 6 Department of Physiology, University of Oklahoma Health Sciences Center, Oklahoma City, OK 73104, USA; 7 Oklahoma City Veterans Affairs Medical Center, Oklahoma City, OK 73104, USA

## Abstract

**Background:**

Influenza is a significant cause of morbidity and mortality. The recent pandemic of a novel H1N1 influenza virus has stressed the importance of the search for effective treatments for this disease. Essential oils from aromatic plants have been used for a wide variety of applications, such as personal hygiene, therapeutic massage and even medical practice. In this paper, we investigate the potential role of an essential oil in antiviral activity.

**Methods:**

We studied a commercial essential oil blend, On Guard™, and evaluated its ability in modulating influenza virus, A/PR8/34 (PR8), infection in Madin-Darby canine kidney (MDCK) cells. Influenza virus was first incubated with the essential oil and infectivity in MDCK cells was quantified by fluorescent focus assay (FFA). In order to determine the mechanism of effects of essential oil in viral infection inhibition, we measured hemagglutination (HA) activity, binding and internalization of untreated and oil-treated virus in MDCK cells by flow cytometry and immunofluorescence microscopy. In addition, the effect of oil treatment on viral transcription and translation were assayed by relative end-point RT-PCR and western blot analysis.

**Results:**

Influenza virus infectivity was suppressed by essential oil treatment in a dose-dependent manner; the number of nascent viral particles released from MDCK cells was reduced by 90% and by 40% when virus was treated with 1:4,000 and 1:6,000 dilutions of the oil, respectively. Oil treatment of the virus also decreased direct infection of the cells as the number of infected MDCK cells decreased by 90% and 45% when virus was treated with 1:2,000 and 1:3,000 dilutions of the oil, respectively. This was not due to a decrease in HA activity, as HA was preserved despite oil treatment. In addition, oil treatment did not affect virus binding or internalization in MDCK cells. These effects did not appear to be due to cytotoxicity of the oil as MDCK cell viability was only seen with concentrations of oil that were 2 to 6 times greater than the doses that inhibited viral infectivity. RT-PCR and western blotting demonstrated that oil treatment of the virus inhibited viral NP and NS1 protein, but not mRNA expression.

**Conclusions:**

An essential oil blend significantly attenuates influenza virus PR8 infectivity *in vitro *without affecting viral binding or cellular internalization in MDCK cells. Oil treated virus continued to express viral mRNAs but had minimal expression of viral proteins, suggesting that the antiviral effect may be due to inhibition of viral protein translation.

## Background

The recent pandemic of a novel H1N1 influenza emphasizes the urgency of identifying effective approaches to prevent viral infection. Between 1990 and 1999 in the United States, non-pandemic influenza A virus (IAV) infected 5-20% of people and caused approximately 36,000 deaths and 226,000 hospitalizations annually [1,2]. IAV infection is initiated with a binding of the viral hemagglutinin (HA) to sialic acid on the cell surface; and virus particles are internalized through receptor-mediated endocytosis. While in the endosome, viral HA protein is activated and the virus fuses with endosomal membranes. After fusion, IAV shuts off host cell protein synthesis and cell replication. As a result, infected cells die by apoptosis or cytolysis.

Essential oils have been used for aromatherapy, massage therapy, emotional health, personal care, nutritional supplements, or cleaning for many years. The modern use of essential oils has grown rapidly as health scientists and medical practitioners have found scientific evidence for the benefits of this therapy. In Japan, Perillae Herba (a leaf of *Perilla frutescens*) has been prescribed to treat depression. It has been shown that *l*-Perillaldehyde, a major component in the essential oil containing in Perillae Herba, is responsible for the antidepressant-like activity through stimulation of the olfactory nerve [3]. Essential oil from *Boswellia carteri *and *Boswellia serrata *have been used for the treatment of rheumatoid arthritis and other inflammatory diseases in traditional medicine for many years [4,5]. Studies showed that frankincense oil derived from *Boswellia *species possess anti-inflammatory activity though inhibition of immune cytokines production and leukocyte infiltration [6-8]. In addition, many other essential oils used in aromatherapy have medicinal properties including antiseptic properties [9], and mood enhancing effects [10,11].

Distinctive chemical components of plants protect them from insects, bacteria or viruses that cause diseases. Essential oils prepared from plants, therefore, might be effective in protecting humans from viral infection. In addition to their intrinsic benefits to plants and as fragrances for people, essential oils have been used throughout history in many cultures for their medicinal and therapeutic benefits. They were first used in ancient Egypt for treatment of various illnesses and other physical and spiritual needs. Borrowing from the Egyptians, the Greeks, Romans, Indians, Persians, as well as Chinese began to refine distillation methods for extracting oils from aromatic plants and have used them extensively in medical practice for diverse purposes, such as promoting wellness, enhancing personal hygiene, and in therapeutic massage and aromatherapy. They have also been used as a beauty treatment, in food preparation, and in religious ceremonies.

In this study, we evaluated the effect of a commercially available essential oil blend, On Guard™, on influenza virus A/PR/8/34 (PR8) infection in Madin-Darby canine kidney (MDCK) cells. This oil blend combines a mixture of wild orange, clove, cinnamon, eucalyptus and rosemary. The mechanism of the oil-mediated inhibition of viral infectivity was also investigated.

## Methods

### Reagents and chemicals

Cell culture medium MEM and DMEM/F12 (1:1), fetal calf serum (FCS), and penicillin-streptomycin were purchased from Invitrogen (Carlsbad, CA). Trypan blue was purchased from Sigma (St. Louis, MO). On Guard™ protective blend oil was obtained from dōTERRA International (Orem, UT). Mouse anti-IAV nucleoprotein (NP) monoclonal antibody was purchased from Abcam (Cambridge, MA). Alexa Fluro 488-conjugated goat anti-mouse IgG was purchased from Molecular Probes (Eugene, OR). Goat anti-IAV NS1 polyclonal antibody was purchased from Santa Cruz (Santa Cruz, CA).

### Preparation of influenza virus stock and cell culture

Influenza A/PR/8/34 (PR8), a laboratory adapted H1N1 IAV strain, was passaged in MDCK cells. MDCK cells were cultured in DMEM supplemented with 10% FCS. Viruses were grown in MDCK cells in DMEM/F12 with ITS+ (containing insulin, human transferrin, and selenous acid; BD Biosciences, Franklin Lakes, NJ) and trypsin (0.5 μg/ml), harvested at 72 h postinfection and titered by plaque assay in MDCK cells. There was no detectable endotoxin in the final viral preparations used in the experiments as determined by limulus amebocyte lysate assay (Cambrex, Walkersville, MD). The assay has a detection limit of 0.1 EU/ml or approximately 20 pg/ml lipopolysaccharide (LPS).

For essential oil-treated influenza virus, PR8 virus was mixed with serial dilutions of the essential oil and incubated at room temperature for 2 h. PR8 virus mixed and incubated in 1-1,000 dilutions of canola oil was used as a control. Oil-treated and untreated IAV was stored at -80°C until use.

### Fluorescent focus assay (FFA)

To determine the release of IAV infectious particles from oil-treated and untreated IAV-infected MDCK cells, cells were grown to confluence in 24-well plates and oil-treated and untreated PR8 virus was added at the multiplicity of infection (MOI) of 1 and incubated at 37°C in a cell incubator. At 48 h after infection, 50 μl aliquots of cell culture supernatants were collected and transferred to 96-well plates containing confluent MDCK cells. The supernatants were serially diluted with PBS containing 0.6 mM CaCl_2 _and 0.5 mM MgCl_2 _(CaMg-PBS) and incubated with the MDCK cells for 45 minutes at 37°C, followed by washing of the cells three times in serum-free DMEM/F12 with ITS+ containing 1% penicillin and streptomycin. The cells were maintained in DMEM/F12 with ITS+.

After 6 h at 37°C, the MDCK monolayers were subsequently washed three times with PBS and fixed with 4% paraformaldehyde for 10 min at 4°C followed by permeabilization with 2% Triton X-100 for 10 min in room temperature. The cell monolayers were then labeled by incubating with a monoclonal antibody (Abcam, Cambridge, MA) against the IAV NP in Staining Buffer (PBS with 0.1%BSA, 1% heat-inactivated human serum, 0.02%NaN_3_) for 30 min at 4°C. Following washes with PBS, Alexa Fluor 488-conjugated goat anti-mouse IgG was added to detect antibody binding to the IAV NPs. Initially, various dilutions of virus were used to find the dose yielding ~50 fluorescent foci per high powered (×40) field. These foci appeared to be single infected cells in general.

### Cell viability analysis

To determine the MDCK cell viability following essential oil treatment, cells were seeded in 24-well tissue culture plates at the density of 5 × 10^4 ^cells/well in 500 μl growth medium for adherence. Aliquots of 500 μl varying dilutions from 1:3,000 to 1:18,000 (v/v) of the oil blend in cell growth media were added to each well in triplicate. Cell viability was determined at 7 h and 24 h following protective blend oil exposure using a Trypan blue (0.4% w/v) exclusion assay. Cell viability was counted using a hemocytometer, and expressed as the percentage of trypan blue positive cells over the total number (stained + unstained) of cells.

### Hemagglutinin titration of influenza virus

For HA assay, viruses were serially 2-fold diluted in a 96-well plate with CaMg-PBS and an equal volume of 0.5% human red blood cells were added. The plates were kept at 4°C for 60 minutes and agglutination was determined visually.

### Flow cytometry analysis and confocal microscopy of IAV binding and internalization

To prepare the cells for flow cytometry and confocal microscopy to examine viral binding and internalization, MDCK cells in exponential growth phase were trypsinized and resuspended in DMEM. Cells (5 × 10^5^) in 100 μl were exposed to PR8 at an MOI of 50, and incubated at 4°C for 30 minutes to allow virus binding. A negative control was performed by exposing cells to an equal volume of sterile virus-free buffer. For oil-treated virus, PR8 was incubated in the protective essential oil at a dilution of 1 to 4,000 for 2 h prior to the exposure. For virus internalization, cells were further incubated with virus at 37°C for 30 minutes. Surface associated virus was removed by incubating the cells in sialidase from *Clostridium perfringens *(Sigma, St Louis, MO) at 40 mU in 200 μl of CMS/Ac, pH 5.5 (150 mM NaCl, 10 mM CaCl_2 _and 0.5 mM MaCl_2_) for 1 h at 37°C with gentle rocking. Following the incubation with virus and/or sialidase, the cells were washed in triplicate to remove unbound virus and then incubated for 20 minutes in 100 μl stain buffer (BD Biosciences, San Jose, CA) with 5% FCS containing 1 μl canine IgG solution as an FcR blocker. After being treated with Perm/Wash Buffer (BD Biosciences) for 10 minutes, the cells were stained with an anti-IAV NP monoclonal antibody followed by incubation with an Alexa Fluor 488-conjugated goat anti-mouse IgG for 40 minutes. The cells were fixed by incubating on ice with 250 μl Cytofix (BD Biosciences) and then washed in triplicate. For FACS analysis, the cells were resuspended in 250 μl Stain Buffer; and single cell suspensions were prepared using a mesh filter. A FACScan cytometer, BD Biosciences LSR II, was used to assess PR8 binding and internalization. A minimum of twenty thousand events were counted for each sample. Analytical gates were set so that ≤1% of negative control cells exceeded the gate. The percentage of cells exceeding the gate was used to determine virus binding and internalization. For confocal microscopy, the cells were resuspended in 200 μl of Stain Buffer and mounted on microscope slides using acrylic-based mounting medium containing DAPI (Invitrogen).

### Measurement of viral mRNA expression by relative end-point RT-PCR

MDCK cells were exposed to oil-treated or untreated PR8 at an MOI of 32. For mock infection, cells were exposed to sterile virus-free buffer. After 18 h, total RNA from MDCK cells was extracted using TRIzol (Invitrogen, Carlsbad, CA). The quantity and quality of the isolated RNA was determined spectrophometrically and by formaldehyde agarose gel electrophoresis, respectively. An equal amount (1 μg) of the total RNA was reverse transcribed for first-strand cDNA synthesis. Then an aliquot of the cDNA was subjected to 30 cycles of PCR amplification (95°C, 30 sec.; 55°C, 1 min.; 72°C, 1 min.) in the presence of primer pairs targeting NP, NS1 or GAPDH. The following primer sequences were used: NP sense 5'-AGGACAAGAGCTCTTGTTCG-3', and anti-sense 5'-CTCTTGTGTGCTGGATTCTC-3'; NS1 sense 5'-GACCAAGAACTGAGTGATGC-3', and anti-sense 5'-TGACCTAGCTGTTCTCGCC-3'; GAPDH sense 5'-TGAAGGTCGGAGTCAACGGATTTGGT-3', and anti-sense 5'-CATGTGGGCCATGAGGTCCACCAC-3'. Aliquots of 25 μl PCR products were electrophoresed on 1% agarose gels and visualized by ethidium bromide staining. DNA bands were imaged and quantified using ImageQuant 5.0 software (GE Healthcare, Piscataway, NJ). Levels of NP and NS1 were normalized to the corresponding GAPDH levels for each sample.

### Viral NS1 protein determination by immunoblotting

MDCK cells were exposed to oil-treated or untreated PR8 at an MOI of 32. For mock infection, cells were exposed to an equal volume of virus-free buffer. Cells were harvested at 18 h following infection by lysing the cells with RIPA buffer (150 mM NaCl; 50 mM Tris, pH 8.0; 10 mM EDTA, NaF, and sodium pyrophosphate; 1% NP-40; 0.5% sodium deoxycholate; 0.1% SDS; 10 μg of leupeptin/ml). Cell homogenates were clarified by centrifugation at 4°C; and 20 to 30 μg of the lysates were separated on 4-15% gradient SDS-PAGE gels. Proteins were then transferred onto nitrocellulose membranes. Following blocking with 3% non fat milk plus 1% BSA, the membranes were incubated anti-IAV NS1 or anti-β-actin (Abcam) antibody overnight followed by incubation with horseradish peroxidase-conjugated rabbit anti-goat IgG (Cell Signaling Technology). Target protein binding was detected using a chemilluminescent reagent (Pierce Biotechnology, Rockford, IL), and visualized by the Syngene G:box Bioimaging System and GeneTools software (Syngene, Frederick, MD).

### Statistical analysis

Results are reported as the mean ± standard error of mean (SEM) of at least three replicate experiments. All statistical analysis was carried out with GraphPad Instat 3. Statistical significance was determined by one-way ANOVA with Student-Newman-Keuls post hoc correction for multiple comparisons. Statistical significant was considered when p < 0.05.

## Results

### Protective essential oil suppressed progeny virus production

The effect of oil treatment on viral infectivity was first determined by measuring the release of nascent viral particles following infection of MDCK cells with untreated, oil treated, or control oil treated virus for 48 h.

**Figure 1:**
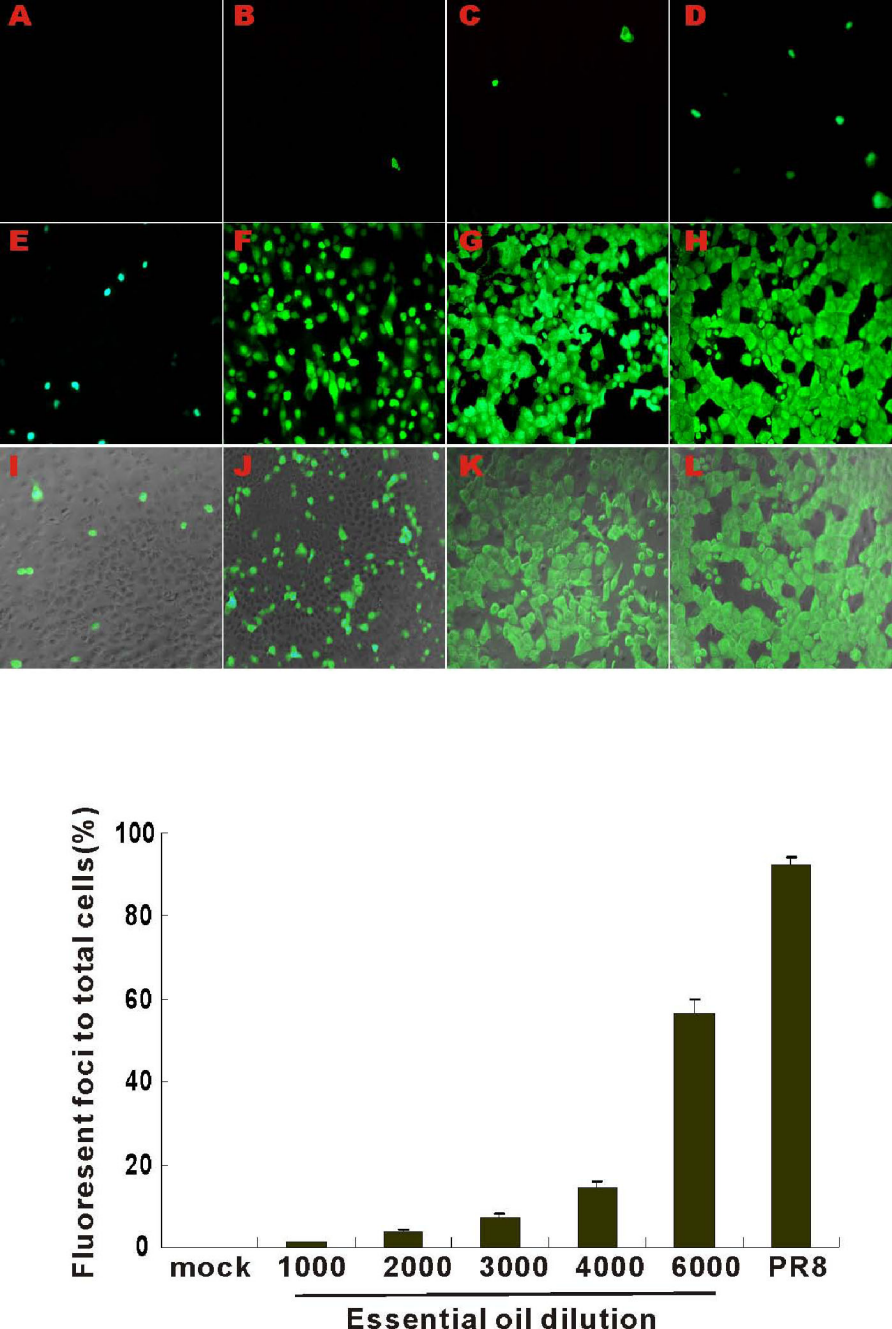
**Effect of oil treatment on progeny virus production by PR8 as measured by Fluorescent focus assay (FFA)**. After MDCK cells in 24-well plates were infected with oil-treated and untreated virus for 48 h, five microliters of supernatants were removed, serially diluted and added to confluent MDCK cells in 96-well plates. After incubation for 7 h, IAV nucleoprotein (NP) was detected using an Alexa Fluor 488 (green) labeled antibody. Panels: (A) MDCK cells unexposed to virus, but stained with anti-NP antibody. Panels (B-F) MDCK cells exposed to PR8 treated with different dilutions of essential oil: (B) 1:1,000 (C) 1:2,000 (D) 1:3,000 (E) 1:4,000 (F) 1:6,000, (G) untreated PR8, (H) PR8 treated with control oil at a 1:1,000 dilution. Panels I-L were fluorescence images merged with corresponding brightfield images to show MDCK cell morphology: (I) PR8 treated with essential oil 1:4,000, (J) PR8 treated with essential oil 1:6,000, (K) untreated PR8, (L) PR8 treated with control oil 1:1,000 (merge). Bottom panel: Infectivity as reflected by the percentage of cells in which IAV NP was detected. The results represent the mean ± SEM from three independent experiments.

Quantification of infectious particles produced in virus-exposed MDCK supernatant was done by transfer of the infected cell supernatant to a separate culture of MDCK cells followed by Fluorescent focus assay (FFA). The fluorescent foci were counted by fluorescent microscopy. There was no detectable green fluorescent signal in cells exposed to virus-free diluents (Figure 1A, top panels). Oil treatment inhibited nascent PR8 production and release into infected supernatants in a dose dependent manner (Figure 1). Virus treatment with control canola oil (1:1,000) had no significant effect on viral particle production (Figure 1G and 1H, top panels). IAV pretreatment with protective oil decreased production of viral particles (Figure 1B-F, top panels).

Virus treated with a 1:4,000 dilution of protective essential oil decreased the infectious particle number by 90% (Figure 1, bottom panel). This did not appear to be due to a toxic effect of the oil on MDCK cells. Because addition of treated virus to MDCK cells resulted in a further dilution of the oil in the media, cells were actually exposed to protective oil at a concentration 1,500 times lower than that used to treat the virus. For example, treatment of the virus with a 1 to 3,000 dilution of oil resulted in exposure of the MDCK cells to a cellular exposure to a 1 to 4,500,000 dilution of oil. This is far below the minimal concentration that caused any detectable cellular cytotoxicity (1: 3,000; Figure 5). Thus, it appeared that the protective oil inhibited IAV PR8 viral production in MDCK cells was not due to non-specific cytotoxicity.

### Protective essential oil suppresses virus infection

**Figure 2:**
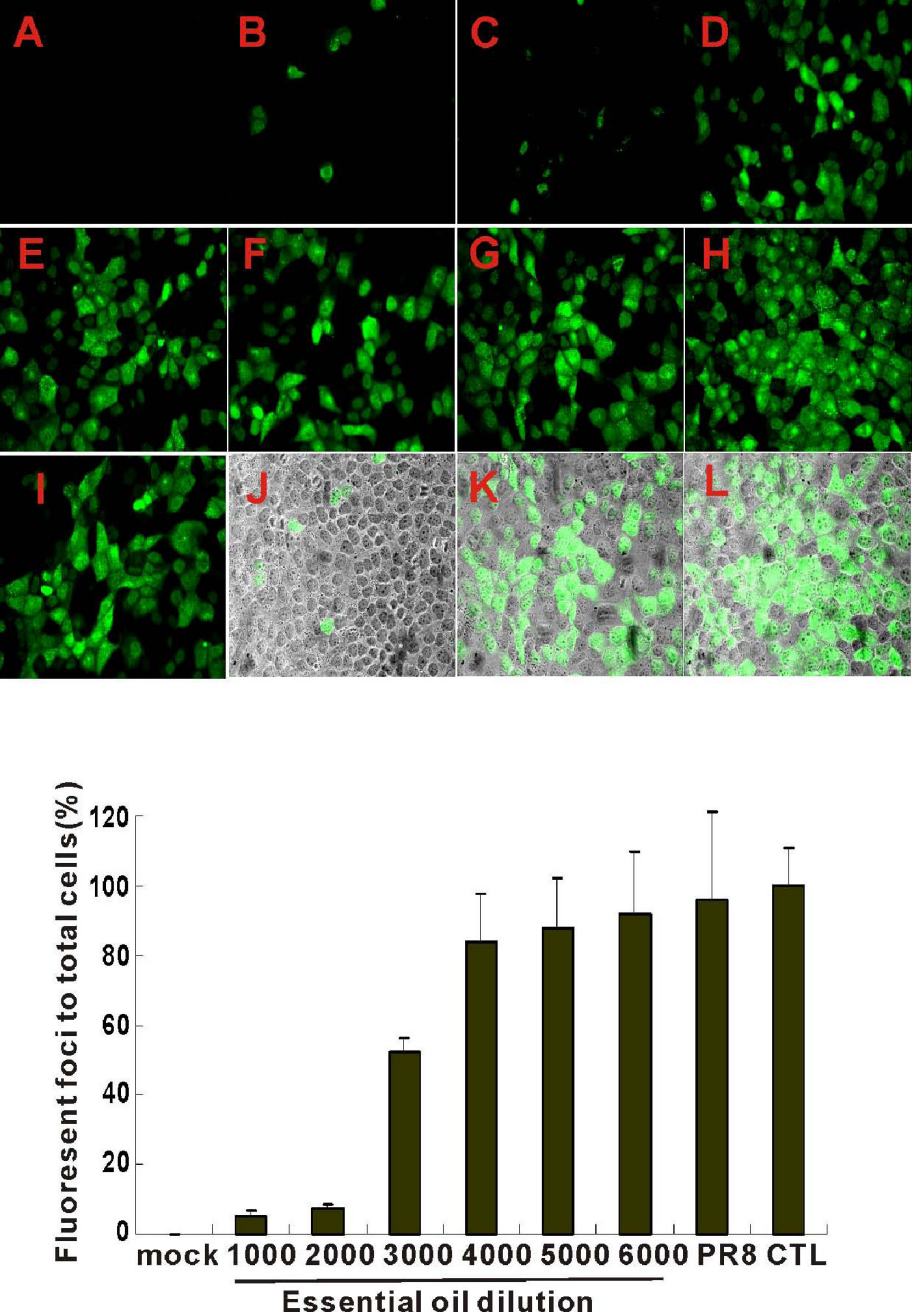
**Effect of essential oil on the first cycle of PR8 infection as determined by FFA**. MDCK cells in 96-well plates were infected by oil-treated virus for 7 h, and viral NP was detected using an Alexa Fluor 488 (green) labeled antibody. Panels: (A) MDCK cells unexposed to virus, but stained with anti-NP antibody. Panels B-F cells exposed to PR8 treated with dilutions of essential oil (B) 1:1,000 (C) 1:2,000 (D) 1:3,000 (E) 1:4,000 (F) 1:5,000, (G) 1:6,000, (H) untreated PR8, (I) PR8 treated with control oil 1:1,000. Panels I-L were fluorescence images merged with corresponding brightfield images to show MDCK cell morphology (J) PR8 treated with essential oil 1:1,000, (K) PR8 treated with essential oil 1:6,000, (L) untreated PR8 (merge). Bottom panel: PR8 first cycle NP production as depicted by the percentage of cells containing detectable NP at 7 h after infection. The results represent the mean ± SEM from three independent experiments.

To determine whether protective oil directly inhibited the first cycle of IAV infection, oil-treated PR8 was added to MDCK cells and direct FFA assay of the infected cells was performed. Fluorescent foci of infected cells were easily detectable after 7 h of exposure to untreated or control canola oil (1:1,000) treated PR8. The nascent produced nucleoprotein (NP) by FFA was significantly decreased by virus treatment by oil at concentrations greater than 1 to 3,000 (Figure 2B-D, top panels). Treatment of PR8 with a 1 to 3,000 dilution of oil decreased the number of infected MDCK cells by 50% (Figure 2, bottom panel). As the oil was further diluted, the final oil dilutions applied to MDCK cells during exposure to virus were 3 fold higher (more dilute) than those used to treat virus in this experiment. For example, treatment of virus with a 1 to 3,000 dilution of oil resulted in MDCK cell exposure of 1 to 9,000. Thus, the protective essential oil significantly inhibited IAV PR8 viral protein production in MDCK cells at concentrations that did not appear to be directly toxic to cells.

### Effect of oil treatment on HA activity

To determine whether the effect of protective oil on virus infectivity was due to alterations in virus particle integrity, the effect of oil treatment on HA activity was assessed. HA activity was measured in untreated PR8 virus or virus treated with various dilutions of protective oil or control oil for 24 h, 48 h and 72 h. Although HA activity decreased with time for all treated and mock treated viruses, there was no significant effect of oil treatment on HA activity (Table 1) at several concentrations that decrease viral infectivity and progeny virus production. There was a modest effect of a 1 to 1,000 dilution on HA activity after 72 h oil treatment. This effect was not seen when dilutions greater than 1:1,000 were used, even with 72 h of exposure. This data shows that the effect of oil treatment on infectivity and viral progeny production was not due to inhibition of HA activity.

**Table 1:** HA titration comparison of essential oil-treated to untreated influenza virus*.

		Essential Oil Dilution						
			
Time (h) after oil treatment	No Oil	1/1000	1/2000	1/3000	1/4000	1/5000	1/6000	Control Oil
24	5	5	5	5	5	5	5	5
48	4	4	4	4	4	4	4	4
72	3	2	3	3	3	3	3	3

*HA titer showing by the last well forming hemagglutination.

### Binding and internalization of virus in MDCK cells

**Figure 3:**
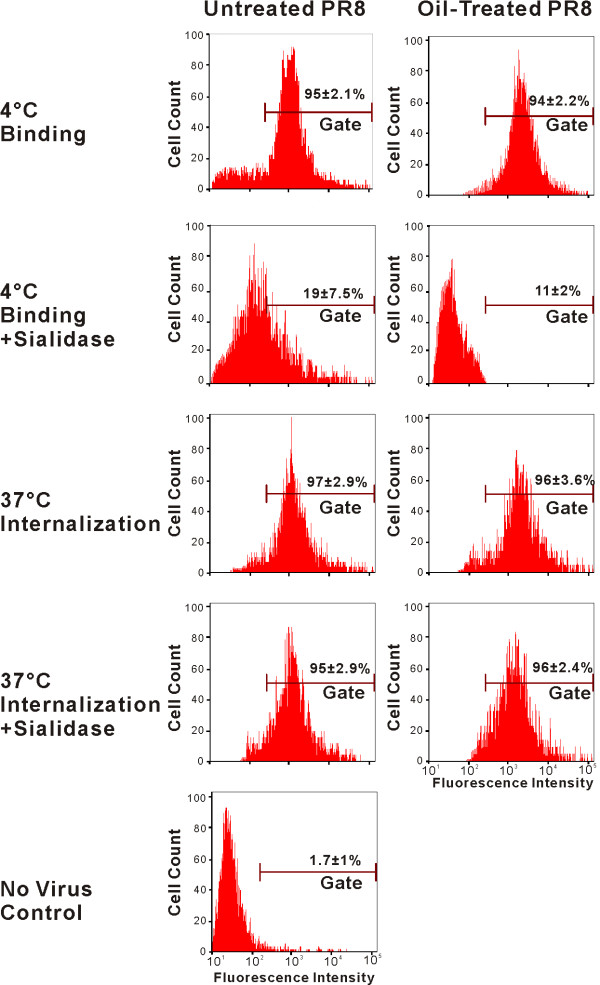
**Essential oil dose not block IAV PR8 binding and entry to MDCK cells as determined by flow cytometry**. MDCK cells were exposed to oil-treated (1:4,000 dilution) and untreated PR8. Viruses were allowed to bind to the cells at 4°C for 30 minutes, or allowed to bind and then internalize at 37°C for 30 minutes. Following the incubation of selected samples with sialidase treatment, viral NP was stained with the fluorescent dye Alexa Fluor 488. The percentage of cells exceeding the analytical gate was used to determine viral binding and internalization. The data are representative of three separate experiments.

In order to examine the mechanism of inhibition of PR8 by oil treatment, the effect of this treatment on virus internalization was studied. To this end, we developed a flow cytometry (FACS) internalization assay. Virus was detected using an Alexa Fluor 488 labeled secondary antibody against monoclonal antibody to PR8 NP. At 4°C, virions attach to the host cell surface, but internalization does not occur. Sialidase is then used to remove the bound, but not internalized virus. Cell associated virus present after sialidase treatment is presumed to be internalized, and this was indeed confirmed by confocal microscopy (see below). FACS assay demonstrated as expected that PR8 bound to receptors at the cell surface but was not internalized at 4°C as the cell-associated fluorescence of stained influenza virus was removed by sialidase (Figure 3, left panels). At 37°C, PR8 internalization occurred, as cell-associated fluorescence was not removed by sialidase treatment (Figure 3, left panels). As determined by the FACS assay, oil treatment did not affect virus binding to MDCK cells. Also, internalization was not detectably affected by oil treatment of PR8 as determined by the amount of sialidase-resistant cell associated fluorescence after incubation of virus exposed cells at 37°C (Figure 3E, L, right panels).

**Figure 4:**
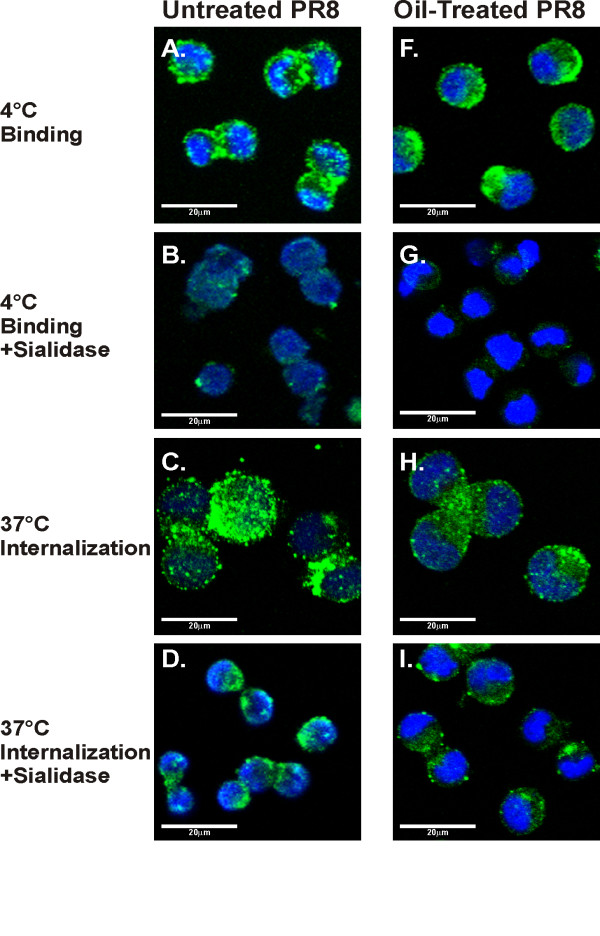
**Essential oil dose not block IAV PR8 binding and entry in MDCK cells as confirmed by confocal imaging**. Binding and internalization of oil treated (1:4,000 dilution) and untreated PR8 virus to MDCK cells was examined as described in Figure 3. Following infection, NP was stained with the fluorescent dye Alexa Fluor 488. Cell nuclei were stained with DAPI (purple).

Results from confocal microscopy confirmed the results of the flow cytometry assay for virus binding and internalization (Figure 4). At 4°C, viruses remained on the cell surface as shown as a green fluorescent ring around the cell membrane (Figure 4, panel A and F). In contrast, the viruses were present in the cytoplasm after incubation at 37°C (Figure 4, panel C and H). Cell-associated influenza virus in cells incubated at 4°C was removed by sialidase, but not after incubation at 37°C confirming that sialidase treatment removed the surface bound, but not internalized, virus (Figure 4, panel B, G, D and I). There was no apparent effect of oil treatment on binding and internalization of PR8. Together, these data demonstrate that oil treatment does not appear to inhibit IAV infectivity and progeny production by alteration of virus binding and internalization.

### Effect of essential oil on cell viability

To determine if essential oil inhibited virus infectivity and progeny production by direct cellular cytotoxic effects, the viability of MDCK cells was measured after incubation with media in the presence or absence of oil. Viability was determined by morphological examination and trypan blue exclusion. Dilutions of oil corresponding to final concentrations of the cells were also used. This was to account for the fact that after oil exposure of the virus, addition of treated virus to MDCK cells resulted in a further dilution of the oil in the media. Therefore, dilutions of protective oil (1:3,000 to 1:18,000) in cell growth media, corresponding to working dilutions for cells in FFA assay, were added to each well in triplicate. For these experiments, two incubation times were used. Seven-hour incubation was used to duplicate the exposure of MDCK cells to oil during the assay for the effect of oil on the first cycle of virus infection (see Figure 2). Twenty-four hours of exposure were used to duplicate the oil exposure during the assay for the effect of oil treatment on viral progeny production (see Figure 1).

Morphologically, oil-treated cells did not show signs of death at 7 h. At 24 h of exposure, MDCK cells remained attached to the bottom of plates and did not show noticeable morphological alterations at the dilution up to 1:3,000.

**Figure 5:**
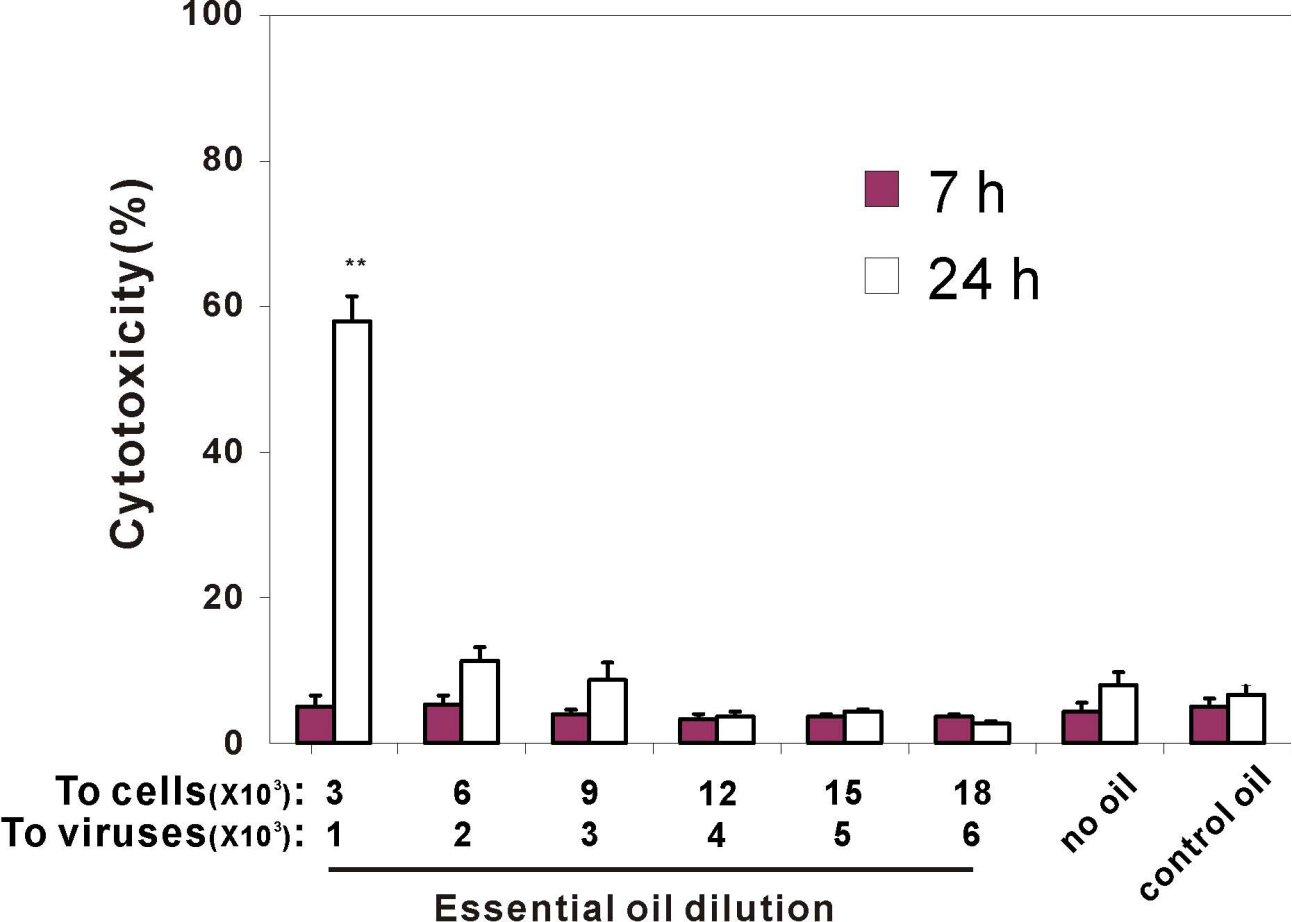
**Effects of essential oil treatment on PR8 production are not due to cytotoxicity**. Cell viability was determined using trypan blue exclusion at 7 and 24 h of essential oil exposure. Data were presented as the mean ± SEM from at least 3 independent experiments. Means were compared to data from the control oil group. **P < 0.01.

Cell viability by trypan blue exclusion was also determined at 7 and 24 h following exposure to the essential oil blend. At all concentrations of oil, all MDCK cells were alive after 7 h of incubation. After 24 h, cell viability was not significantly affected by the increasing concentrations of protective blend oil up to a 1: 3,000 dilution (Figure 5). At this concentration about 60% of the cells were dead. Control oil (canola oil, 1:1,000) did not cause any cell death after 24 h. As there was no effect of any concentration the oil on cell viability at 7 h the effect of oil treatment on the first cycle of viral infection does not appear to be due to cytotoxicity. Also, as the oil concentrations causing cytoxicity after 24 h of incubation were much greater than those that inhibited viral progeny production, it appears that this effect of oil treatment is not due to cytotoxic effects of the oil.

### MDCK cells infected by oil-treated virus express viral mRNA, but minimal amounts of protein

**Figure 6:**
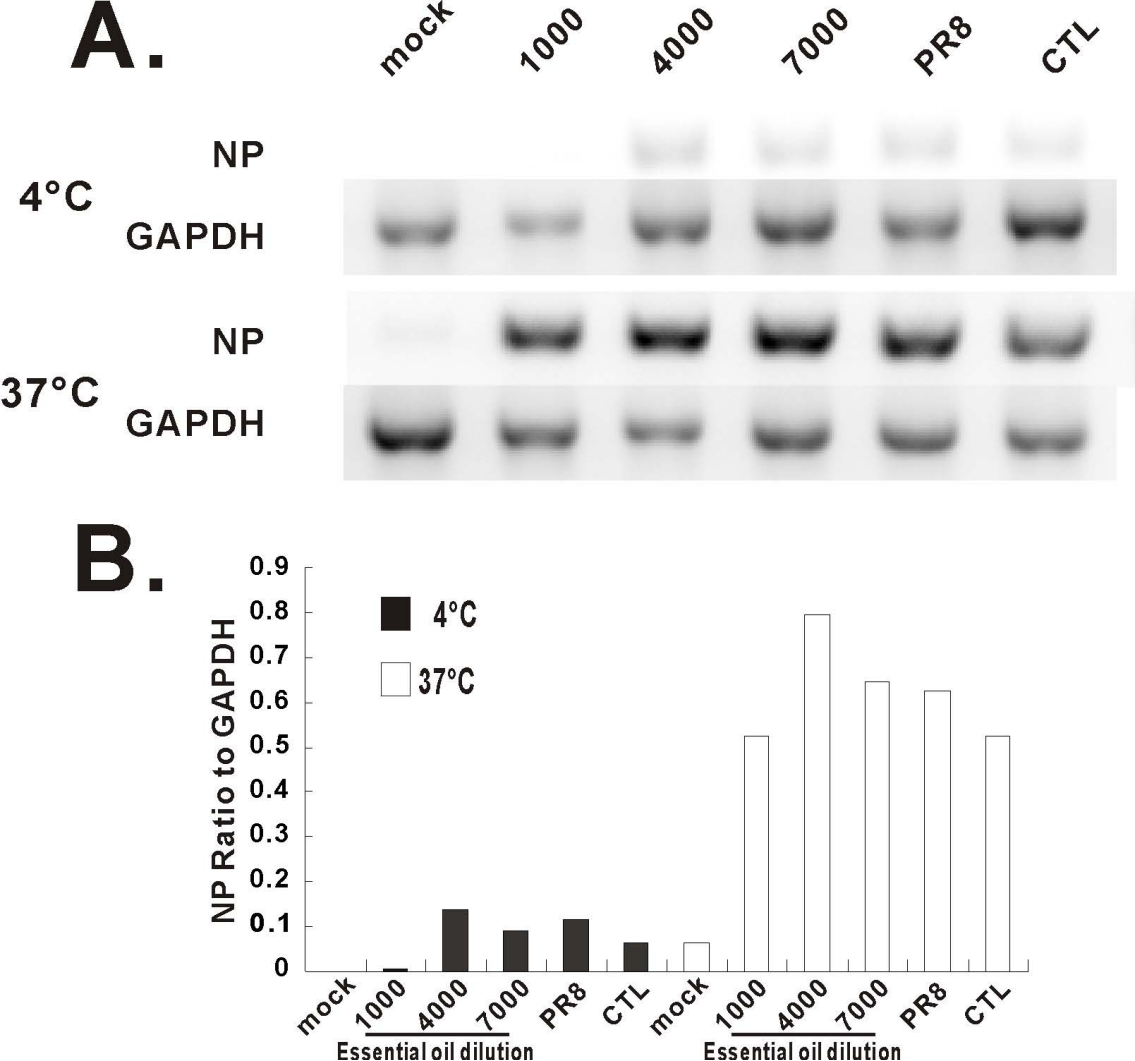
**Essential oil treatment does not inhibit PR8 NP mRNA expression in MDCK cells**. After infection of MDCK cells with oil-treated (1:4,000 dilution) and untreated PR8 virus at 4°C or 37°C for 18 h, total RNA was extracted and PR8 NP mRNA expression was assessed by relative end-point RT-PCR (A). Transcript levels of NP normalized relative to the constitutively expressed GAPDH gene (B). The data are representative of three separate experiments.

We next sought to determine whether the effects of oil treatment on viral infectivity and progeny production could be due to inhibition of viral gene expression at the transcriptional level. Endogenous mRNA levels of viral NP were determined using relative end-point RT-PCR. As expected, when cells were exposed to virus at 4°C, no viral NP RNA was detected, consistent with virus binding, but failing to internalize under these conditions. At 37°C, RNA expression of NP was detected in cells infected with both oil-treated virus and untreated PR8 virus. NP mRNA expression levels were similar whether oil-treated or untreated virus was used (Figure 6). This finding suggests that the decrease in NP protein expression seen with oil treatment (see Figure 2) was likely due to inhibition of viral mRNA transcription.

**Figure 7:**
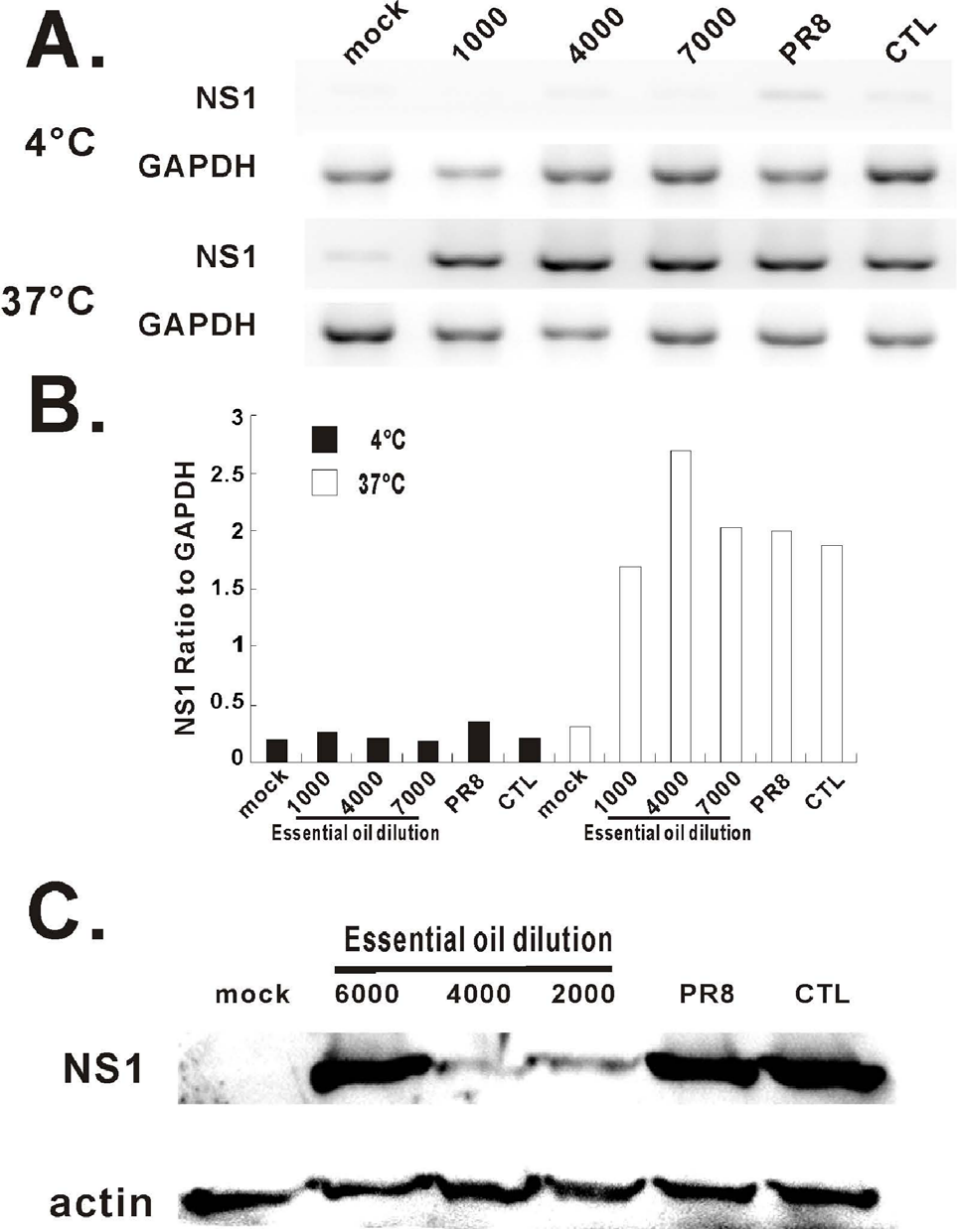
**MDCK cells infected by oil-treated virus express viral NS1 mRNA, but minimal amounts of NS1 protein**. After infection of MDCK cells with oil-treated (1:4,000 dilution) and untreated PR8 virus at 4°C and 37°C for 18 h. Total RNA was extracted from cells and PR8 NS1 mRNA expression was assessed by relative end-point RT-PCR (A). Transcript levels of NS1 normalized relative to the constitutively expressed GAPDH gene (B). Western blot was used to determine NS1 protein expression in the infected cells (C). The membranes were probed with anti-NS1 or anti-actin antibodies. The data depicted are representative of three separate experiments.

To confirm whether oil treatment inhibited viral protein but not mRNA production, we also measured mRNA and protein expression levels of another viral protein, NS1, in control and oil treated virus-exposed cells. As with viral NP, mRNA expression of NS1 was not affected by oil treatment of PR8 (Figure 7A). In contrast, NS1 protein expression was significantly decreased by treatment of PR8 with essential oil. (Figure 7B). Based on the above results, inhibition of viral progeny production and infection by essential oil is likely due to inhibition of viral protein synthesis.

## Discussion

In order to develop novel therapies, many plant extracts have been tested for antiviral activity. Over the last decade, laboratory research has found that many essential oils diminish viral infectivity. For example, *Melissa officinalis *oil decreases infectivity of enveloped herpesviruses [12]. Essential oils from hyssop, thyme or ginger have inhibitory effects on herpes simplex virus type 2 (HSV-2) *in vitro *as determined by plaque assay in RC-37 cells [13].

With regard to IAV, *Melaleuca alternifolia *essential oil has antiviral activity against influenza PR8 virus and that antiviral activity has been principally attributed to a specific component, terpinen-4-ol [14]. The anti-IAV activity is manifested by inhibition of virus-induced cytopathogenicity. Also, a polyphenol rich extract (CYSTUS052) from the Mediterranean plant *Cistus incanus *exerts potent anti-influenza virus activity in A549 or MDCK cell cultures infected with IAV. On a molecular basis the protective effect of CYSTUS052 appears to be mainly due to binding of the polymeric polyphenol components of the extract to the virus surface, thereby inhibiting binding of the hemagglutinin to cellular receptors [15]. *Echinacea purpurea *extract has activity against highly pathogenic avian influenza virus, via inhibition of cell receptor binding activity of the virus [16].

Replication of the virus RNA genome in infected cells was found to be constantly suppressed in the presence of the *Agrimonia pilosa *extract. The data suggests that the extract exerts an antiviral effect on multiple rounds of the influenza infection cycle, primarily the initial step of the virus life cycle, but activity at later stages, including replication and transcription, cannot be ruled out [17]. Study of catechins in green tea on influenza virus suggests that the antiviral effect of catechins on influenza virus is mediated not only by specific interaction with HA, but altering the physical properties of viral membrane [18]. Here, we report that a blend of essential oil attenuates IAV infection in MDCK cells. Our work shows that the mechanism of antiviral activity is due to inhibition of viral protein synthesis. This mechanism of inhibition is similar to that of trans-cinnamaldehyde (CA), one of the principal constituents of essential oil derived from Cinnamomi cortex. This extract inhibits the growth of influenza PR8 virus *in vitro *and inhibited viral protein, but not mRNA synthesis [19]. In this study, they were also able to demonstrate a protective effect of CA in PR8 infected mice. Thus, the inhibitory activity of plant extracts appears to be at multiple stages of viral infection and proliferation.

Some traditional herbal medicines have been used for the treatment of various diseases for more than 2,000 years. Recently some have been officially approved for clinical use. For example, aspirin was originally made from powder of the bark and leaves of the willow tree to help heal headaches, pains and fevers back in Hippocrates days. Currently, 148 Japanese kampo medicines have now formally been approved by the Ministry of Health, Labor and Welfare of Japan for use in clinics. In addition, several kampo were shown to have immuno-modulatory and anti-viral effects both *in vitro *and *in vivo *[20]. With more understanding of their antiviral mechanisms, more traditional medicines will be utilized for clinical pharmaceutical purposes and novel drug discovery.

The lack of toxicity and potent specific viral inhibitory activity suggest essential oil may be helpful as a possible antiviral drug for control and treatment of influenza virus infection. It could potentially be used as a non-toxic way to cleanse surfaces, or dispersed to eliminate aerosolized virus particles in confined areas. Since the oil is currently used as a food supplement, oral administration, once the pharmacokinetics are determined, may provide therapeutic benefit during infection.

## Conclusions

We have shown that virus treated with protective essential oil significantly decreased both the number of released viral particles from infected MDCK cells, and infected cells by FFA assay. Also, oil treated virus had the same ability to bind to and internalize in MDCK cells compared with untreated virus. MDCK cells infected by oil-treated virus express viral mRNA, but minimal amounts of protein. Taken together, we found an essential oil blend notably attenuates influenza virus PR8 infection *in vitro *via inhibition of viral protein synthesis at the post-transcription level. The lack of toxicity and potent specific inhibition ability make the essential oil a possible antiviral drug for influenza virus proliferation control and treatment.

## Competing interests

None of the authors have competing financial interests to disclose. All authors have no links to doTERRA International, producer of the On Guard™ blend oil.

## Authors' contributions

SW and WW designed the study and analyzed the data. WW drafted the manuscript. KBP, JPM, HL and SW collected the data and participated to their interpretation. JLB assisted with preparation of the figures. All authors read and approved the final manuscript.

## Pre-publication history

The pre-publication history for this paper can be accessed here:

http://www.biomedcentral.com/1472-6882/10/69/prepub
